# Early computed tomography coronary angiography in patients with suspected acute coronary syndrome: randomised controlled trial

**DOI:** 10.1136/bmj.o438

**Published:** 2022-02-21

In this paper by Gray and colleagues (*BMJ* 2021;374:n2106, doi:10.1136/bmj.n2106, published 29 September 2021), two research team members were inadvertently missed in the article’s submission. The article and PDF will be updated in due course.

